# The Biological Mechanisms and Clinical Roles of RNA-Binding Proteins in Cardiovascular Diseases

**DOI:** 10.3390/biom14091056

**Published:** 2024-08-25

**Authors:** Lizhu Lin, Jiemei Chu, Sanqi An, Xinli Liu, Runxian Tan

**Affiliations:** 1 Department of Anaesthesiology, The First People’s Hospital of Qinzhou, The Tenth Affiliated Hospital of Guangxi Medical University, Qinzhou 535000, China; lizhulin529@gmail.com; 2 Life Sciences Institute, Guangxi Medical University, Nanning 530021, China; jmchu@gxmu.edu.cn (J.C.); ansq@mail2.sysu.edu.cn (S.A.); 3 Department of Laboratory Medicine, The First People’s Hospital of Qinzhou, The Tenth Affiliated Hospital of Guangxi Medical University, Qinzhou 535000, China

**Keywords:** RNA-binding proteins (RBPs), cardiovascular diseases (CVDs), myocardial injury, RNA alternative splicing, therapeutic targets, biomarkers

## Abstract

RNA-binding proteins (RBPs) have pivotal roles in cardiovascular biology, influencing various molecular mechanisms underlying cardiovascular diseases (CVDs). This review explores the significant roles of RBPs, focusing on their regulation of RNA alternative splicing, polyadenylation, and RNA editing, and their impact on CVD pathogenesis. For instance, RBPs are crucial in myocardial injury, contributing to disease progression and repair mechanisms. This review systematically analyzes the roles of RBPs in myocardial injury, arrhythmias, myocardial infarction, and heart failure, revealing intricate interactions that influence disease outcomes. Furthermore, the potential of RBPs as therapeutic targets for cardiovascular dysfunction is explored, highlighting the advances in drug development and clinical research. This review also discusses the emerging role of RBPs as biomarkers for cardiovascular diseases, offering insights into their diagnostic and prognostic potential. Despite significant progress, current research faces several limitations, which are critically examined. Finally, this review identifies the major challenges and outlines future research directions to advance the understanding and application of RBPs in cardiovascular medicine.

## 1. Introduction

Cardiovascular diseases (CVDs) are the leading cause of death worldwide, with ischemic heart disease and stroke being the most prevalent types, responsible for over 80% of these fatalities. These conditions affect both the systemic and pulmonary circulations and can impact all cardiac chambers. Globally, they result in an approximate mortality rate of 235 deaths per 100,000 individuals [1]. 

Cardiovascular disorders can arise independently or as a consequence of vascular conditions, consistently ranking as a leading cause of death. Pulmonary hypertension (PH) and atherosclerosis are predominant issues within the pulmonary and systemic vascular systems, respectively. Specifically, pulmonary arterial hypertension (PAH), a common subtype of PH, has the potential to result in right ventricular enlargement, cardiac insufficiency, and ultimately, fatal outcomes. Atherosclerosis, characterized by arterial stiffening and constriction due to neointima development, immune cell infiltration, and lipid accumulation, significantly contributes to high rates of heart-related fatalities and health issues [2]. Common cardiovascular conditions include heart failure (HF), myocardial infarction (MI), heart muscle injury, and arrhythmias. HF manifests as changes in the heart’s dimensions, configuration, and function, reducing its pumping capacity. MI, caused by coronary artery blockage, results in reduced blood supply to the heart muscle and eventual cardiomyocyte death. Arrhythmias frequently occur following myocardial infarction. Although reperfusion therapy is essential for MI patients, it paradoxically carries the risk of myocardial damage via ischemia-reperfusion injury [3]. 

Cardiovascular conditions, whether arising from genetic predispositions or environmental influences, significantly alter gene expression patterns. This intricate process is tightly regulated, with chromatin architecture playing a crucial role in controlling gene accessibility, alongside the protein degradation systems. RNA-binding proteins (RBPs) are essential in modulating RNA transcripts, governing their diverse roles throughout various stages of RNA processing. RBPs are involved in splicing, maturation, localization, stability, and translation of RNA transcripts. Since 2014 [4], review articles have highlighted the critical roles of RBPs in heart development, covering every stage from cardiac formation to morphogenesis and maturation. This foundational research has laid the groundwork for extensive studies on the relationship between RBPs and cardiovascular development, function, and disease. Recent research [5] has further underscored the importance of RBPs, particularly in the context of congenital heart disease (CHD), the most common birth defect affecting over 1.35 million newborns worldwide.

RBPs are produced in various tissues and released at specific developmental stages or in response to cellular signals. Their expression and activity are tightly regulated, ensuring precise temporal and spatial control of RNA metabolism. In the heart, RBPs are expressed both during embryonic development and in the adult myocardium, where they play crucial roles in maintaining normal cardiac function. The timing and localization of RBP expression are critical, as they modulate key processes such as RNA splicing, stability, and translation, which are essential for proper cardiac development and response to injury. These processes are indispensable for normal heart development and response to damage. Based on these fundamental functions, RBPs also play a critical regulatory role in a wide range of cellular activities within the nucleus and cytoplasm. Defined by their RNA-binding domains (RBDs), RBPs have the ability to recognize and interact with specific RNA sequences or structures, thereby influencing the fate and function of the bound RNA, as well as the expression profiles of associated genes [6]. RBPMS, a key RNA-binding protein with multiple splicing isoforms, plays a pivotal role in heart development by modulating mRNA splicing. Recent studies [7] have demonstrated that knocking down RBPMS in human induced pluripotent stem cell-derived cardiomyocytes significantly impairs their contractile function. The versatility of RBPs is demonstrated by their ability to interact with various RNA species, including mRNA exons, introns, untranslated regions (UTRs), and non-coding RNAs, which are crucial for the modulation of gene expression [8,9]. Another recent study [10] revealed that knocking out YTHDF1 in adult mouse cardiomyocytes results in cardiac hypertrophy, fibrosis, and dysfunction, underscoring the critical role of specific RBPs in cardiac disease and the maintenance of heart function. This finding also opens new avenues for research into cardiac dysfunction. RBPs are integral to translation initiation and termination processes, regulating ribosome assembly on target mRNAs to influence translation rate and efficiency, thereby altering protein synthesis [11,12]. The complexity of RNA–protein interactions is demonstrated by the diverse mechanisms and structures RBPs use to bind and regulate RNA [13]. Studies [14] have shown that RBPs play a critical role in the survival and proliferation of cardiomyocytes, regulate cardiac regeneration and repair capacity, and are essential in preserving cardiac structure and function.

This review explores the impact of RBPs in cardiovascular science, with a focus on the mechanistic links between these proteins and heart disease. Pioneering studies conducted several years ago revealed the crucial role of RBPs in cardiovascular function and dysfunction, and research in this area continues to deepen. In recent years, additional RBPs have been identified as playing key roles in the development of the cardiovascular system, with their dysregulation linked to various diseases and their outcomes. This review also highlights the latest breakthroughs regarding RBPs in myocardial injury, arrhythmias, and congestive heart failure, and examines therapeutic advancements targeting RBPs as well as their emerging role as diagnostic biomarkers. These scientific discoveries not only offer new insights into the mechanisms of cardiovascular diseases but also pave the way for the development of RBP-targeted therapies.

## 2. Function of RNA-Binding Proteins in Cardiovascular Biology through RNA Regulation

**Figure 1:**
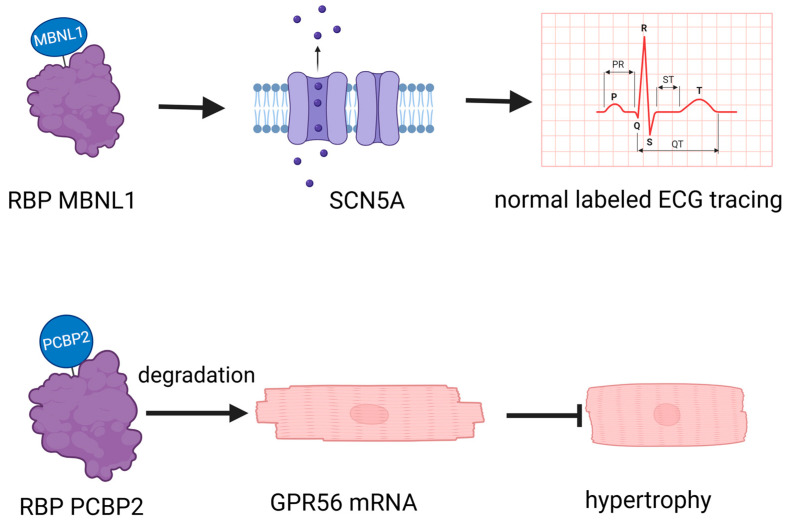
RNA-binding proteins (RBPs) have a crucial role in cardiovascular biology. MBNL1: muscleblind-like 1, a crucial regulator of cardiomyocyte maturity, controls the transition between proliferative and hypertrophic growth, and is essential for stabilizing gene expression networks that maintain the differentiated state of adult heart cells. PCBP2: Poly(rC)-binding protein 2, a key post-transcriptional and translational regulator, plays a crucial role in inhibiting cardiac hypertrophy by stabilizing mRNA and repressing the expression of target genes such as GPR56 in heart tissue. GPR56: G protein-coupled receptor 56, a critical transmembrane receptor, is involved in the regulation of cell–cell interactions, myelin sheath formation, and the development of the central nervous and hematopoietic systems. GPR56 plays an essential role in inhibiting cardiac hypertrophy by modulating gene expression and interacting with various ligands.

RBPs play a crucial role in cardiovascular biology, operating through diverse mechanisms that profoundly impact heart and vascular health (Table 1). For instance, the RBP muscleblind-like 1 (MBNL1) regulates the expression of SCN5A [15], crucial for cardiac electrical activity. Another example is Poly(rC) binding protein 2 (PCBP2), which promotes the degradation of G protein-coupled receptor 56 (GPR56) mRNA in cardiomyocytes, thereby inhibiting angiotensin II-induced hypertrophy [16] (Figure 1). This illustrates the multifaceted ways in which RBPs influence cardiac function, encompassing gene expression regulation, mRNA stability, and degradation. While Potel KN’s review focuses specifically on RBPs in diabetic cardiomyopathy [17], their pivotal roles extend beyond this condition. They intricately influence the complexities of the vascular network, playing a significant part in both the emergence and progression of cardiovascular health issues [18]. RBPs intricately affect the vascular network, significantly contributing to the onset and progression of cardiovascular issues [18]. The diverse mechanisms through which RBPs operate in cardiovascular biology underscore their critical role in maintaining heart and vascular health, highlighting their potential as therapeutic targets for various cardiovascular conditions.

### 2.1. RNA-Binding Proteins Affect Cardiovascular Diseases through Polyadenylation

**Figure 2:**
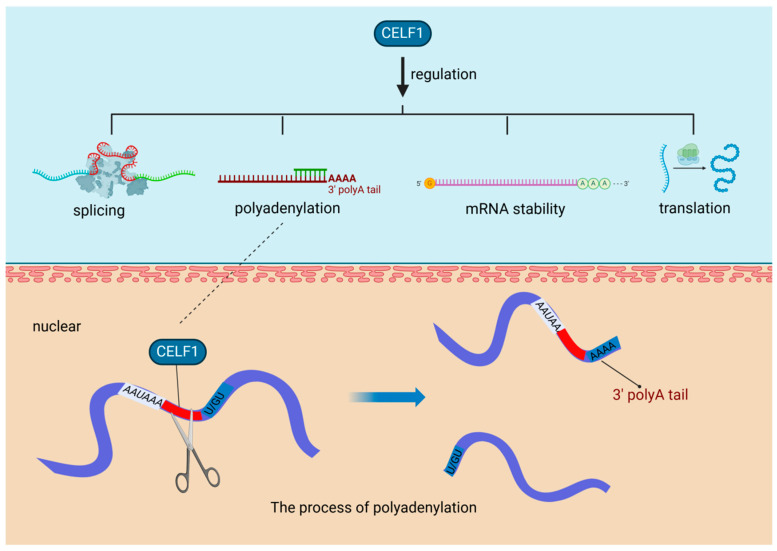
RNA-Binding proteins affect cardiovascular diseases through polyadenylation. In diabetic hearts, the RNA-binding protein CUG-BP (CELF1) is upregulated, playing a crucial role in key processes like alternative splicing and polyadenylation, the latter involving cleavage and poly(A) tail addition to mRNA transcripts. CUG-BP (CELF1): CUGBP elav-like family member 1, a multifunctional RNA-binding protein, is crucial for the regulation of mRNA splicing, stability, and translation in various tissues, including the myocardium, playing an essential role in myofibril organization, cell proliferation, and morphogenesis during cardiac development.

In diabetic hearts, a notable increase in the expression of the RBP CUG-BP, also recognized as CUGBP elav-like family member 1 (CELF1), has been observed. This evolutionarily conserved RBP plays a vital role in various key cellular processes, including the regulation of alternative splicing, mRNA polyadenylation, mRNA stability, and translation efficiency [20]. Polyadenylation, a critical mechanism influenced by CELF1, is a two-step process. Initially, it involves the cleavage of the transcript at a specific point between the AAUAAA consensus sequence and a variable U/GU-rich region downstream of the cleavage site. Following this, the poly(A) polymerase appends a poly(A) tail to the cleaved transcript [30] (Figure 2). The heightened presence of CELF1 under diabetic conditions highlights the intricate relationship between metabolic disorders and gene expression regulation. CELF1′s involvement in polyadenylation is essential for preserving mRNA integrity and facilitating efficient translation, which significantly impacts cellular protein synthesis. In the context of cardiovascular diseases, especially those exacerbated by diabetes, the role of RBPs such as CELF1 becomes more pronounced. They can modify the expression of genes critical for cardiac function, potentially leading to alterations in the heart’s structure and function. Understanding the regulation of RBPs like CELF1 in various pathological conditions could pave the way for innovative therapeutic strategies to address the cardiovascular challenges posed by diabetes.

### 2.2. RNA-Binding Proteins Influence Cardiovascular Diseases by Regulating RNA Alternative Splicing

**Figure 3:**
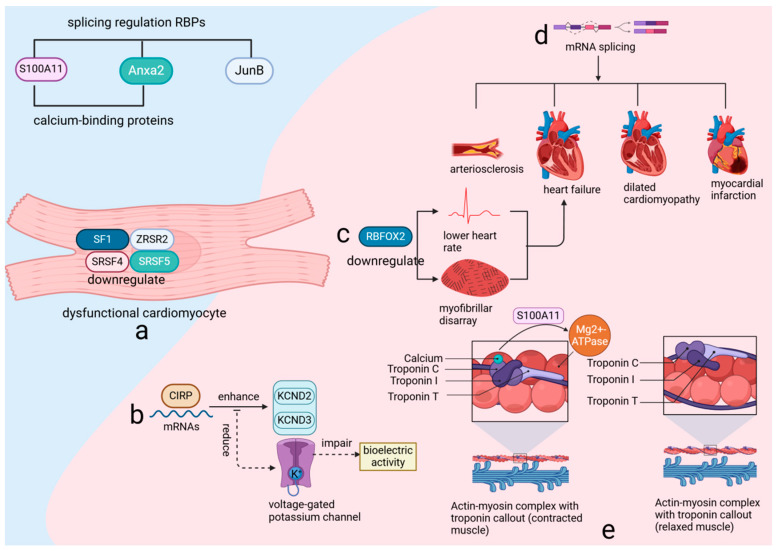
RNA-binding proteins (RBPs) utilize RNA-binding domains to regulate gene expression through various post-transcriptional mechanisms. (**a**) the expression of critical splicing factors such as SF1, ZRSR2, SRSF4, and SRSF5 is downregulated in dysfunctional cardiomyocytes. (**b**) CIRP binding to heart RNAs boosts KCND2 and KCND3 translation, enhancing potassium channel activity. (**c**) RBFOX2 downregulation causes heart issues, leading to failure. (**d**) Variable splicing links to various heart diseases. (**e**) S100A11, Anxa2, and JunB regulate splicing and affect muscle activity with calcium. SF1: Splicing Factor 1, a key RNA splicing factor, plays a crucial role in maintaining cardiac function by regulating mRNA splicing, and its downregulation in dysfunctional cardiomyocytes contributes to impaired cardiac function and disease progression. SF1 is essential for the proper expression of cardiac genes and the maintenance of cellular homeostasis, influencing processes such as cell survival, differentiation, and response to stress. ZRSR2: Zinc finger CCCH-type, RNA-binding motif and serine/arginine-rich 2, a critical RNA splicing factor, plays an essential role in the regulation of mRNA splicing and is crucial for maintaining cardiac function. Its downregulation in dysfunctional cardiomyocytes impairs cardiac reprogramming and enhances the conversion of cardiac fibroblasts to induced cardiomyocytes (iCMs), highlighting its importance in cellular differentiation and heart disease progression. SRSF4: Serine/arginine-rich splicing factor 4, a crucial RNA-binding protein involved in the regulation of alternative splicing, plays an essential role in maintaining cardiac function. Its downregulation in dysfunctional cardiomyocytes leads to cardiac hypertrophy, diastolic dysfunction, and abnormal repolarization, highlighting its significance in the progression of heart disease. SRSF5: Serine/arginine-rich splicing factor 5, a glucose-inducible RNA-binding protein, plays a crucial role in heart development by regulating the alternative splicing of key cardiac structural genes. CIRP: Cold-inducible RNA-binding protein, a crucial regulator of RNA stability and translation, plays a vital role in maintaining cardiac bioelectric activity by facilitating the translation of key potassium channel subunits, KCND2 and KCND3. KCND2: Potassium voltage-gated channel subfamily D member 2, a crucial component of the transient outward potassium current (I_TO) in the heart, plays a vital role in cardiac repolarization and electrical stability. KCND3: Potassium voltage-gated channel subfamily D member 3, a key subunit of the A-type potassium current (I_TO), is essential for the proper electrical function of cardiac and neuronal cells by contributing to the regulation of action potential duration and excitability. RBFOX2: RNA-binding protein fox-1 homolog 2, a key regulator of alternative splicing, plays a crucial role in heart development and function, particularly in the context of diabetic cardiomyopathy and cardiovascular disease. S100A11: S100 calcium-binding protein A11, a key regulator in calcium-dependent signaling pathways, plays a crucial role in modulating actin dynamics and myosin ATPase activity, essential for cellular function and heart physiology. ANXA2: Annexin A2, a calcium-binding protein involved in various cellular processes, plays a crucial role in regulating autophagy, inflammation, and angiogenesis, particularly in response to cardiac injury and stress. JunB: Jun B proto-oncogene, a critical transcription factor and member of the AP-1 complex, plays a vital role in regulating gene expression in response to cellular stress, inflammation, and differentiation, significantly impacting cardiovascular function and development.

RBPs are crucial regulators of gene expression, influencing post-transcriptional processes such as alternative splicing and RNA degradation. Their role in cardiovascular health and disease is well documented, with studies underscoring their significance [18,31]. In adult hearts, the splicing patterns of RBPs are intimately connected to cardiomyocyte functionality. For example, the downregulation of key splicing factors—including splicing factor 1 (SF1), zinc finger CCCH-type, RNA-binding motif and serine/arginine-rich 2 (ZRSR2), serine/arginine-rich splicing factor 4 (SRSF4), and serine/arginine-rich splicing factor 5 (SRSF5)—in dysfunctional cardiomyocytes highlights their essential role in maintaining cardiac function (Figure 3a). Additionally, the interaction of the cold-inducible RNA-binding protein (CIRP) with mature RNAs in the heart facilitates the translation of potassium voltage-gated channel subfamily D members, KCND2 and KCND3. Disruption of this process results in reduced performance of voltage-gated potassium channels and impaired bioelectric activity [32] (Figure 3b), thereby emphasizing the delicate balance that RBPs sustain in cardiac cells.

RNA-binding protein fox-1 homolog 2 (RBFOX2), a critical RNA-binding protein (RBP), is renowned for its regulatory influence on numerous genes essential for cardiac function through alternative splicing. Experimental evidence from various animal models has consistently shown that reduced expression levels of RBFOX2 are associated with a decreased heart rate and structural disarray of myofibrils, and can lead to heart failure [26,27] (Figure 3c). Additionally, recent research has highlighted the increased mRNA splicing variability and abnormal expression of RBPs during myocardial ischemia-reperfusion injury (MIRI), emphasizing the importance of alternative splicing in this pathological context [33]. Furthermore, the correlation between mRNA splicing variability and a range of cardiovascular diseases, including arteriosclerosis, heart failure, dilated cardiomyopathy, and myocardial infarction [34] (Figure 3d), underscores the extensive impact of RBPs on cardiac integrity.

The RNA-binding protein-regulated alternative splicing events (RBP–RASE) covariance regulatory network suggests a potential regulatory effect of RBPs on variable splicing. Among these, S100 calcium-binding protein A11 (S100A11), annexin A2 (Anxa2), and jun B proto-oncogene (JunB) are key players in this regulatory mechanism. Both S100A11 and Anxa2, recognized as calcium-binding proteins capable of forming tetrameric structures, either mixed or identical, are integral to the regulatory process. S100A11, a member of the S100 family, interacts with actin in the presence of calcium, which is crucial for modulating myosin magnesium ATPase activity [35] (Figure 3e). This interaction underscores the importance of calcium signaling in cardiac function and highlights the role RBPs play in this process. Expanding the perspective, it is evident that over 95% of multi-exon genes in higher eukaryotic organisms, including humans, are subject to alternative splicing [36]. This process, governed by RBPs that recognize specific sequences, is essential for creating a diverse proteome and is believed to be a driving force behind speciation and the complexity of phenotypes [37]. These findings underscore the critical role of RBPs not only in maintaining cardiac function but also in contributing to biological diversity and complexity.

### 2.3. RNA-Binding Proteins Influence Cardiovascular Diseases by Regulating RNA Modification

The regulation of RNA modifications by RBPs is currently a prominent research focus, and numerous studies have demonstrated that RBPs can regulate the development of cardiovascular diseases through RNA modification. Fat mass and obesity-associated protein (FTO), an N6-methyladenosine (m^6^A) demethylase, and methyltransferase-like 3 (METTL3), an m^6^A methyltransferase, are pivotal regulators in the methylation landscape associated with heart diseases.

**Figure 4:**
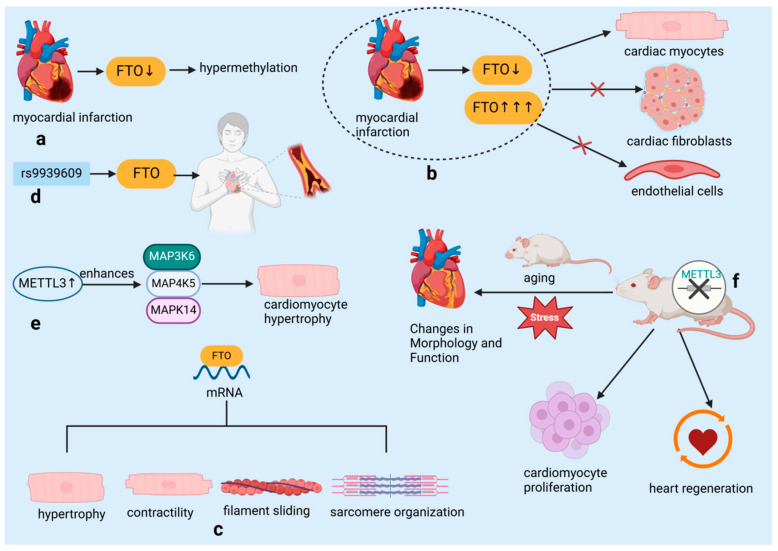
RNA-binding proteins influence cardiovascular diseases by regulating RNA modification. (**a**) Hypermethylation due to downregulation of m^6^A eraser protein FTO post-myocardial infarction. (**b**) FTO expression and hypoxia-induced downregulation limited to cardiac myocytes. (**c**) FTO demethylation targets identified in mRNA transcripts related to hypertrophy, contractility, filament sliding, and sarcomere organization. (**d**) The rs9939609 polymorphism in the FTO gene is associated with an increased risk of coronary heart disease. (**e**) METTL3 overexpression in cardiomyocytes enhances MAP3K6, MAP4K5, and MAPK14 levels and increases cell size. (**f**) Cardiomyocyte-specific METTL3 knockout induces morphological and functional changes; knockdown accelerates heart regeneration and increases proliferation. FTO: Fat mass and obesity-associated protein, an essential regulator of RNA methylation, plays a crucial role in modulating cardiac function and protecting against myocardial ischemia-reperfusion injury by influencing gene expression stability and cellular responses such as apoptosis and inflammation. METTL3: Methyltransferase-like 3, an essential RNA methyltransferase, plays a crucial role in regulating cardiac function and protecting against myocardial ischemia-reperfusion injury by modulating mitochondrial dynamics and cellular responses, including inflammation and apoptosis. MAP3K6: Mitogen-activated protein kinase kinase kinase 6, a crucial upstream kinase in the MAPK signaling pathway, plays a pivotal role in regulating cellular responses to stress, such as apoptosis and inflammation, by activating downstream kinases including p38 and JNK, which are essential for the progression of myocardial injury and other stress-related cellular processes. MAP4K5: Mitogen-activated protein kinase kinase kinase kinase 5, an upstream regulator in the MAPK signaling cascade, functions by activating MAP3K6 and other MAP3Ks, thereby influencing the activation of key signaling molecules such as p38 and JNK, and contributing to cellular differentiation, stress response, and apoptosis, particularly in the context of cardiac health. MAPK14: Mitogen-activated protein kinase 14, also known as p38α, is a terminal kinase in the MAPK signaling pathway, directly involved in mediating cellular responses to stress, including inflammation, apoptosis, and differentiation, and plays a critical role in the pathogenesis of myocardial ischemia and other cardiovascular diseases.

In early 2019, Mathiyalagan et al. [28] identified a novel mechanism in cardiac tissue following MI. They discovered that the m^6^A demethylase enzyme FTO was downregulated, leading to hypermethylation within the heart (Figure 4a). Intriguingly, high FTO expression and hypoxia-induced FTO downregulation were confined to cardiomyocytes, with no observed changes in cardiac fibroblasts or endothelial cells, underscoring the cell-specific nature of m^6^A regulation (Figure 4b). The research team identified a collection of mRNA transcripts associated with key cardiac functions such as hypertrophy, contractility, sarcomere organization, and filament sliding, which were found to be demethylation targets of FTO (Figure 4c). Their work demonstrated that increasing FTO levels could counteract the hypermethylation of these transcripts, subsequently enhancing cardiac performance post-MI. Additionally, genetic variations in the FTO gene, including the rs9939609 polymorphism, have been correlated with an increased susceptibility to coronary heart disease (CHD), an association that may be modulated by various factors including ethnicity, lifestyle, and environmental conditions [38] (Figure 4d). The demethylase function of FTO is posited to contribute to the maintenance and restoration of cardiac function by influencing the stability and expression levels of mRNAs encoding cardiac contractile proteins [39,40,41].

A recent study by Dorn et al. [29] has shown that the presence of m^6^A methylation on cardiomyocyte mRNA notably increases in response to hypertrophic stimuli. The m^6^A RNA methyltransferase METTL3 is identified as a key player in cardiomyocyte hypertrophy. Experiments in cell broth cultures and living organisms have revealed that overexpression of METTL3 in cardiomyocytes results in elevated levels of mitogen-activated protein kinase kinase kinase 6 (MAP3K6), mitogen-activated protein kinase kinase kinase kinase 5 (MAP4K5), and mitogen-activated protein kinase 14 (MAPK14), which are all part of the mitogen-activated protein kinase pathway. This leads to an enlargement of the cardiomyocytes, thus confirming that elevated METTL3 levels are capable of triggering hypertrophy (Figure 4e). Importantly, despite the increased size of cardiomyocytes, METTL3-overexpressing mice showed no signs of histopathological alterations, indicating that this overexpression does not lead to heart malfunction. Conversely, the targeted deletion of METTL3 in cardiomyocytes resulted in structural and functional alterations in the heart, particularly under conditions of aging and stress, highlighting the importance of RNA methylation in preserving normal heart function (Figure 4f). The significance of METTL3 in heart-related diseases is further supported by its crucial role in the development of the embryonic heart and its link to conditions such as cardiac hypertrophy and myocardial infarction [29]. Suppression of METTL3 has been shown to enhance cardiomyocyte proliferation and expedite heart healing following injury in both neonatal and adult mice, whereas excessive METTL3 is associated with reduced proliferation and hindered heart regeneration in postnatal mice [42] (Figure 4f).

The involvement of FTO and METTL3 in modulating gene expression and protein synthesis within the context of cardiac disorders underscores their significance as prospective therapeutic targets. Anticipating a surge in interest, therapies centered on m^6^A modification for CVDs such as CHD, hypertension, cardiac hypertrophy, and HF are expected to gain prominence. The modulation of m^6^A, facilitated by either the methyltransferase METTL3 or the demethylase FTO, is emerging as a promising avenue for novel treatment strategies. Advances in technology are poised to expand the range of m^6^A-targeted therapies available to cardiologists, potentially revolutionizing cardiovascular disease management. As our understanding of RNA epigenetics deepens, it is anticipated to pave the way for groundbreaking advancements in the molecular therapeutics of CVDs [43].

### 2.4. RNA-Binding Proteins Affect Cardiovascular Diseases through RNA Editing

**Figure 5:**
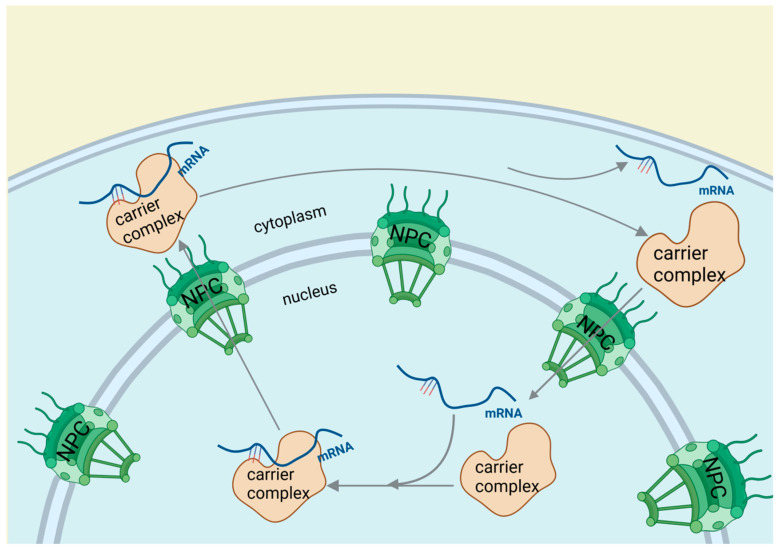
RNA-binding proteins affect cardiovascular diseases through RNA editing. After transcription, splicing, and 3′ end processing, RBPs guide mRNA from the nucleus to the cytoplasm through a three-step process involving cargo-carrier formation, nuclear pore passage, and cytoplasmic mRNA release with carrier recycling.

RBPs are pivotal in modulating mRNA expression and translation, playing a crucial role in the pathogenesis of vascular diseases such as inflammation and atherosclerosis [44]. In eukaryotic organisms, most mRNAs, except for replication-dependent histone mRNAs, acquire a 3′ poly(A) tail of approximately 200 nucleotides. Following transcription, splicing, and 3′ end processing, RBPs assist in the mRNA’s journey from the nucleus to the cytoplasm through several steps: forming a complex with the mRNA in the nucleus, transporting it through the nuclear pore, and releasing it into the cytoplasm, with the recycling of the carrier proteins (Figure 5). RBPs are also instrumental in directing these transcripts to specific cellular compartments and modulating their translation, including mRNA turnover, thereby controlling gene expression and stability. Additionally, RBPs can interact with RNA through chemical modifications at the 5′ cap, 3′ poly(A) tail, and various internal sites, facilitating RNA editing. The primary form of this editing is the conversion of adenosine to inosine, leading to changes in the RNA sequence through mechanisms such as nucleotide insertion, deletion, or substitution. This process enriches the transcriptome and introduces an additional level of gene expression regulation [30,45,46,47,48]. For example, RBPs of the CUG-BP and Elav-like (CELF) family are involved in multiple steps of RNA processing, including pre-mRNA alternative splicing, RNA editing, deadenylation, mRNA degradation, and translation. Dysregulation mediated by CELF is closely related to the pathogenesis of cardiovascular diseases [49].

The significance of RBPs in gene regulation is underscored by their capacity to mediate RNA editing and influence mRNA stability and distribution. Their role in RNA processing and its implications for cardiovascular health is complex and multifaceted. The discovery of RNA editing and its effects on the transcriptome presents new opportunities for understanding and managing cardiovascular diseases. The involvement of RBPs in these processes suggests their potential as therapeutic targets for vascular diseases and atherosclerosis.

## 3. The Role of RNA-Binding Proteins in Cardiovascular Diseases

RBPs are essential regulators in cardiovascular diseases, influencing heart and vascular function through various mechanisms that contribute to the pathogenesis and progression of these conditions (Table 2).

### 3.1. The Role of RNA-Binding Proteins in Heart Muscle Injury

**Figure 6:**
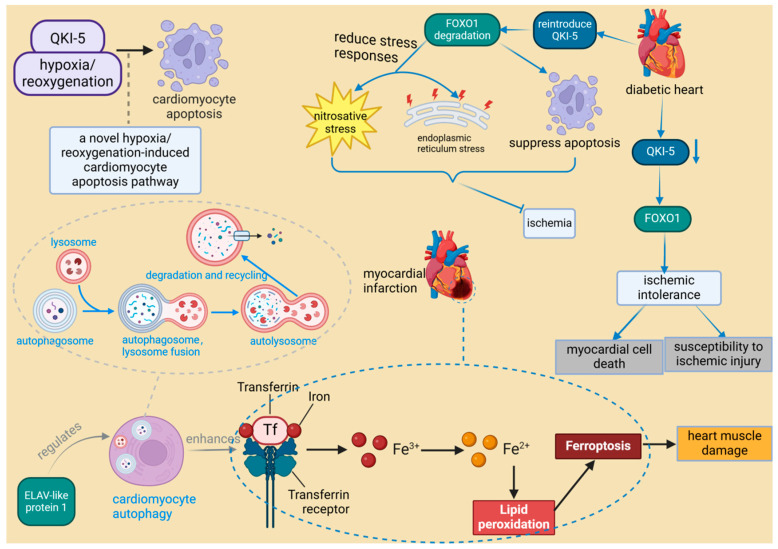
The role of RNA-binding proteins in heart muscle injury. ELAV-like protein 1 enhances ferroptosis in myocardial infarction by regulating autophagy, leading to heart muscle damage. In diabetic hearts, the downregulation of RBP QKI-5 increases vulnerability to ischemia by activating FOXO1. Conversely, reintroduction of QKI-5 inhibits ischemia by reducing apoptosis and mitigating stress responses. ELAVL1: ELAV-like protein 1, a protein that plays a critical role in the progression of inflammation and heart failure, is particularly important under hyperglycemic conditions. It regulates cardiac cell death by modulating the expression of caspase-1 and IL-1 beta in cardiomyocytes, and its expression is influenced by miR-9. QKI-5: Quaking isoform 5, one of the three major alternatively spliced isoforms of the quaking (QKI) RNA-binding protein, plays a specific role in regulating RNA processes crucial for cellular functions. It is noted for its broad biological significance in cardiovascular development and neurological disorders. FOXO1: Forkhead box transcription factor O1, a protein that responds to cellular stimulation and maintains tissue homeostasis by modulating downstream targets including apoptosis- and autophagy-associated genes, anti-oxidative stress enzymes, cell cycle arrest genes, and metabolic and immune regulators. It is involved in multiple signaling pathways through post-transcriptional modifications such as phosphorylation, ubiquitination, acetylation, and others, which activate or inhibit its function.

The growing recognition of RBPs in myocardial injury is exemplified by specific cases such as ELAV-like RNA-binding protein 1 (ELAVL1) and serine/arginine-rich splicing factor 10 (SRSF10). ELAVL1 promotes ferroptosis during myocardial infarction by modulating autophagy, which leads to cardiac muscle damage. In contrast, impaired SRSF10 function has been associated with cardiac rupture [50,51]. Research indicates that SRSF10 interacts with specific circular RNAs (circRNAs) and regulates the splicing of key genes, such as titin (TTN), to maintain the structural and functional integrity of cardiomyocytes [51]. Consequently, it is hypothesized that the impairment of SRSF10 function could disrupt these crucial splicing processes, potentially leading to cardiac rupture. A recent study identified 47 RBPs with significant differential expression following MIRI, with most being upregulated, suggesting a collective response to ischemic stress [35]. Among these RBPs, quaking isoform 5 (QKI-5) stands out as particularly significant. QKI-5 is an RNA-binding protein that plays a critical role in various cellular processes, especially in regulating apoptosis and stress responses in cardiomyocytes. Its function has been validated in vivo. For instance, studies have shown that downregulation of QKI-5 can enhance ischemic tolerance by activating the Forkhead box transcription factor O1 (FOXO1) pathway, thereby increasing cardiomyocyte death and susceptibility to ischemic injury in diabetic hearts [52]. Furthermore, animal experiments have demonstrated that reintroducing QKI-5 can significantly reduce the severity of ischemia-reperfusion injury by inhibiting apoptosis and mitigating nitrosative and endoplasmic reticulum stress responses [52]. Moreover, the role of QKI-5 in the hypoxia/reoxygenation-induced cardiomyocyte apoptosis pathway presents new therapeutic possibilities for ischemic heart disease [80] (Figure 6). QKI-5 is an RNA-binding protein that plays a critical role in various cellular processes, especially in regulating apoptosis and stress responses in cardiomyocytes. Its function has been validated in vivo. Thus, although QKI-5 has shown potential therapeutic value in animal models, its application in humans requires further research and clinical validation. It is noteworthy that QKI-5 is the predominant isoform in human embryonic stem cells (hESCs), cardiogenic progenitor cells, and differentiated cardiomyocytes. Its expression significantly increases during the transition of cardiogenic progenitors to early differentiated cardiomyocytes, suggesting that QKI-5 function is likely more relevant to early cardiogenesis [81].

### 3.2. The Relationship between Arrhythmias and RNA-Binding Proteins

Recent studies on RBPs have revealed their potential in treating cardiac arrhythmias, particularly atrial fibrillation (AF). The discovery of quaking isoform 6 (QKI-6), an RBP, has been associated with improving vascular abnormalities, including AF [53,54]. This is significant because AF can lead to irregular heartbeats, potentially causing serious health complications such as stroke and heart failure. The ability of QKI-6 to improve this condition highlights the crucial role that RBPs might play in the intricate cellular processes regulating heart rhythm. A study by Xie D et al. [55] underscores the importance of RBPs by demonstrating that CIRP plays a critical role in triggering AF. Targeted delivery of CIRP to the atria using adeno-associated virus serotype 9 (AAV9) has been demonstrated to prevent AF in CIRP-deficient rats. These rats also exhibited a decreased atrial effective refractory period (AERP) and action potential duration (APD), increasing their susceptibility to AF due to the upregulation of Kv1.5 and Kv4.2/4.3 channels. The post-transcriptional regulation of these atrial-specific ion channels by CIRP, targeting their 3′ untranslated regions (UTRs), underscores its influence on atrial electrophysiology. Consequently, CIRP emerges as a promising target for intervening in AF. This understanding of the therapeutic potential of RBPs opens a new pathway for arrhythmia treatment, emphasizing the necessity for further investigation into the molecular interplay between RBPs and cardiac health.

### 3.3. The Relationship between Myocardial Infarction and RNA-Binding Proteins

**Figure 7:**
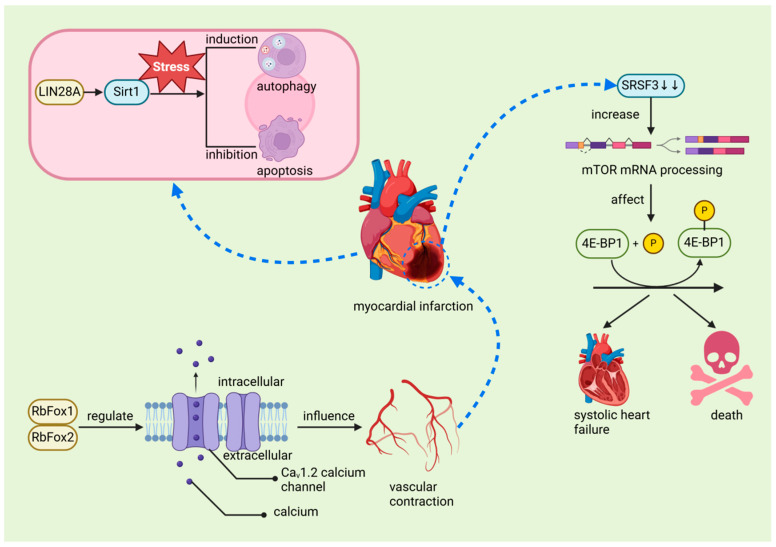
The relationship between myocardial infarction and RNA-binding proteins. In MI, LIN28A activates Sirt1 to induce autophagy and inhibit apoptosis in cardiomyocytes, promoting repair and regeneration. RbFox1 and RbFox2 play a critical role in regulating the alternative splicing of the Ca_v_1.2 calcium channel, which influences calcium influx in smooth muscle cells, leading to abnormal vascular contraction and contributing to myocardial infarction. After MI, reduced expression of SRSF3 results in abnormal mTOR mRNA decapping and alternative splicing, affecting the phosphorylation of 4E-BP1, which can be fatal in severe cases. LIN28A: lin-28 homolog A, a master regulator of cellular metabolism, plays a crucial role in heart development. SIRT1: sirtuin 1, a member of the NAD+-dependent deacetylases family, plays a crucial role in regulating thrombosis, modulating key pathways including endothelial activation, platelet aggregation, and coagulation. RbFox1 and RbFox2: RNA-binding protein fox-1 homolog 1 and 2 regulates the alternative splicing of the Cav1.2 calcium channel, influencing calcium influx and vascular contraction in smooth muscle cells. SRSF3: serine/arginine-rich splicing factor 3, an RNA-binding protein crucial for cardiomyocyte proliferation and cardiac homeostasis, plays a significant role in mRNA splicing and the prevention of systolic heart failure. mTOR: mammalian target of rapamycin, a key regulator of cell growth, proliferation, and survival, plays a pivotal role in cardiac function and homeostasis.

The regenerative capacity of the heart in mature mammals is limited, primarily because cardiomyocytes (CMs) exit the cell cycle shortly after birth. This limitation contrasts with the regenerative abilities observed in juvenile mammals and certain lower vertebrates such as zebrafish and newts. For instance, in zebrafish, cardiomyocyte regeneration relies on extensive changes in the chromatin landscape following injury. Members of the activator protein-1 (AP-1) family, such as JunB and FOS Like 1 (Fosl1), play a crucial role in this process by regulating chromatin accessibility, thereby promoting cardiomyocyte dedifferentiation, proliferation, and eventual protrusion into the damaged area to complete the regeneration process. This mechanism is notably different from the AP-1 response in adult mammalian cardiomyocytes, underscoring the highly regenerative capacity of the zebrafish heart post-injury [82]. These species can effectively regenerate their hearts after ventricular resection or MI [50,52,80], a process closely associated with the regulatory function of RBPs. Studies have demonstrated that Bone Morphogenetic Protein 7 (BMP7) is essential for promoting cardiomyocyte proliferation and cardiac regeneration in both zebrafish and mice [83]. Similarly, mitochondrial fatty acid β-oxidation (FAO) is critical for zebrafish ventricular regeneration, providing the necessary energy for cardiomyocyte proliferation and heart repair. Inhibition of FAO impairs regeneration, a condition that can be partially alleviated by external supplementation of l-carnitine [84]. Furthermore, Histone Deacetylase 1 (Hdac1) plays a pivotal role in zebrafish heart regeneration by regulating key cell cycle components such as Cyclin-Dependent Kinase Inhibitor 1 (p21) and Cell Division Cycle 25 (Cdc25), thereby ensuring proper cardiomyocyte cell cycle progression [85]. Although BMP7, FAO, and Hdac1 are not RBPs, it is plausible that RBPs significantly influence their activity within the broader regulatory network governing heart regeneration [86]. In various animal studies, the role of RBPs in both ischemic and non-ischemic myocardial injury has been widely acknowledged, underscoring their crucial involvement in cardiac repair and their potential to facilitate regeneration. Recent scholarly discussions have further examined the potential therapeutic applications of these proteins [14]. Among these RBPs, RBM15 plays a critical role by enhancing m^6^A levels during MI, thereby regulating the expression and stability of the NEDD8-activating enzyme E1 subunit. Elevated m^6^A levels stabilize the expression of the NEDD8 activating enzyme E1, reduce cardiomyocyte apoptosis, and subsequently improve cardiac function in MI-induced mice. The upregulation of RBM15 and its modulation of m^6^A methylation may represent a protective mechanism that promotes cardiac function recovery, indicating its potential as a therapeutic target for MI [56]. In MI patients, Insulin-Like Growth Factor 2 mRNA-Binding Protein 2 (IGF2BP2) expression is significantly upregulated, which may exacerbate myocardial injury by promoting cardiac remodeling, leading to further deterioration of cardiac function. Furthermore, IGF2BP2 overexpression is associated with abnormal structural changes in cardiomyocytes post-MI, such as mitochondrial fragmentation and sarcomere thinning, suggesting that IGF2BP2 may play a pivotal role in MI pathology and could serve as a key therapeutic target in the future [57]. Similarly, lin-28 homolog A (LIN28A), an evolutionarily conserved RBP, also demonstrates significant therapeutic potential. LIN28A enhances stem cell pluripotency and mediates the transition from self-renewal to differentiation, playing a vital role during MI [87]. During MI, LIN28A activates sirtuin 1 (Sirt1), a stress-responsive NAD(+)-dependent deacetylase, which improves cardiac function by enhancing autophagy and inhibiting cardiomyocyte apoptosis [58] (Figure 7). These findings suggest that LIN28A and similar RBPs hold promise for cardiac repair, particularly in species with limited regenerative capacity. Additionally, RBPs such as RNA-binding protein fox-1 homolog 1 (RbFox1) and RNA-binding protein fox-2 (RbFox2) are essential in regulating the alternative splicing of the Ca_v_1.2 calcium channel, influencing calcium influx and vascular contraction in smooth muscle cells (Figure 7). Under hypoxic conditions, reduced expression of RbFox1 and RbFox2 leads to structural changes in the Ca_v_1.2 channel, including the upregulation of exon 9 * and downregulation of exon 33. These modifications increase the sensitivity of the Ca_v_1.2 channel to calcium channel blockers such as isradipine, which may result in abnormal vascular contraction and heightened risk of MI [60]. Furthermore, circRNAs contribute to the pathogenesis of MI by interacting with RBPs and microRNAs (miRNAs), regulating mRNA transcription, and sponging miRNAs, making them potential biomarkers for MI diagnosis and prognosis [88,89]. Finally, after MI, the reduced expression of the RBP serine/arginine-rich splicing factor 3 (SRSF3) in cardiomyocytes leads to severe systolic dysfunction and death. This process is driven by increased mRNA decapping and alternative splicing of mammalian target of rapamycin (mTOR) mRNA, which affects the phosphorylation of eIF4E-binding protein 1 (4E-BP1) and impacts genes critical for cardiac contraction [61] (Figure 7). These studies, based on cellular and animal models, have unveiled the molecular mechanisms underlying MI and offer new directions for future human therapies. Specifically, these findings identify potential therapeutic targets that could enhance cardiac regeneration, prevent adverse remodeling, and improve cardiac function, thereby potentially enhancing the treatment outcomes for MI patients. However, further research is required to validate these mechanisms in humans and ensure the safety and efficacy of potential therapies. The translation of these findings could lead to more effective therapeutic interventions for MI.

### 3.4. The Interaction between Heart Failure and RNA-Binding Proteins

**Figure 8:**
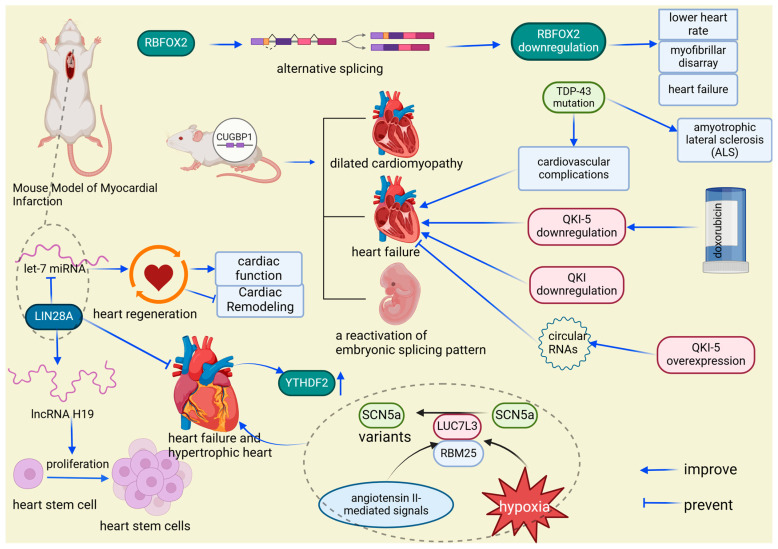
RBP expression variations: implications for cardiac disease. LIN28A enhances stem cell proliferation and heart failure recovery by upregulating lncRNA H19 and interacting with let-7 miRNA, while heart failure is linked to YTHDF2 overexpression and malfunctioning SCN5a splice variants influenced by RBM25 and LUC7L3 in response to cardiac stress. RBPs in cardiac health and disease: a molecular interplay. Mutations in TDP-43 and CUGBP1 overexpression cause cardiovascular issues and heart failure, with reactivation of fetal RBPs and abnormal RBFOX2 and QKI expression impacting heart function, highlighting their roles in cardiac disease, including diabetes and drug-induced conditions. RBFOX2: RNA-binding protein fox-1 homolog 2, a key regulator of alternative splicing, plays a crucial role in heart development and function, particularly in the context of diabetic cardiomyopathy and cardiovascular disease. YTHDF2: YT521-B homology domain family 2, an RNA m^6^A reader protein, regulates cardiomyocyte size and apoptosis, and plays a crucial role in cardiac function, metabolism, and the physiological response to exercise. SCN5a: sodium voltage-gated channel alpha subunit 5, a critical component of cardiac electrical impulse initiation and conduction, plays a crucial role in heart rhythm regulation, particularly in the context of arrhythmias such as long QT syndrome, Brugada syndrome, and sudden infant death syndrome. LUC7L3: LUC7-like 3 pre-mRNA splicing factor, a splicing regulatory protein, indirectly modulates cardiac function by influencing the alternative splicing of key genes such as SCN5A, playing a crucial role in heart rhythm regulation and the pathogenesis of heart failure. RBM25: RNA-binding motif protein 25, a splicing regulatory protein, influences cardiac function by modulating the alternative splicing of critical genes like SCN5a, playing a significant role in the regulation of heart rhythm and contributing to the pathogenesis of heart failure. TDP-43: TAR DNA-binding protein 43, a protein implicated in the formation of neuronal and glial inclusions, plays a crucial role in the pathology of amyotrophic lateral sclerosis (ALS) and is associated with aggregates in skeletal and cardiac muscles in various neuromuscular diseases. CUGBP1: CUG-binding protein 1, a multifunctional RNA-binding protein, is crucial for the regulation of mRNA splicing, stability, and translation in various tissues, including the myocardium, playing an essential role in myofibril organization, cell proliferation, and morphogenesis during cardiac development.

Analysis of the cardiac RBPome reveals significant differences in RNA-binding protein expression among fetal, adult, and heart failure states. The reactivation of fetal-specific RBPs and the dynamic regulation of Cytoplasmic Polyadenylation Element-Binding Protein 4 (Cpeb4) are critical in heart failure, driving pathological cardiac growth and remodeling through targeted mRNA interactions [62,63]. Functionally, long non-coding RNA H19 (lncRNA H19) plays a crucial role in alleviating pathological cardiac hypertrophy by regulating genes involved in cardiac remodeling. LIN28A interacts with lncRNA H19 to enhance its expression and influence stem cell dynamics, potentially mitigating heart failure caused by cardiac hypertrophy [64,65]. Additionally, LIN28A promotes cardiac regeneration through its interaction with lethal-7 microRNA (let-7 miRNA), where inhibiting let-7 activity facilitates cardiac repair, reduces post-infarction remodeling, and improves cardiac function in mice [66,67,68]. CircRNAs, a unique class of long non-coding RNAs characterized by their covalently closed loop structure, also play vital roles in heart failure through interactions with RBPs. For example, circFndc3b interacts with Fused in Sarcoma (FUS) RNA-binding protein to enhance angiogenesis and protect cardiomyocytes, while cTTN1, regulated by RNA-binding motif protein 20 (RBM20), influences the splicing of key cardiac genes, and circ-Foxo3 regulates cardiac aging and function by binding to stress-related proteins [71]. In heart failure, certain proteins, such as YT521-B homology domain family 2 (YTHDF2), are notably upregulated, as observed in tissues affected by human heart failure and hypertrophic mouse hearts [73]. Dysregulation of sodium voltage-gated channel alpha subunit 5 (SCN5a), crucial for conducting electrical impulses in cardiomyocytes, also contributes to heart failure progression [74,90]. Hypoxia and angiotensin II signals activate splicing factors like RNA-binding motif protein 25 (RBM25) and LUC7-like 3 pre-mRNA splicing factor (LUC7L3), leading to the production of dysfunctional SCN5a variants associated with heart failure [74,90,91] (Figure 8). Furthermore, mutations in TAR DNA-binding protein 43 (TDP-43), associated with amyotrophic lateral sclerosis (ALS), have been linked to cardiovascular issues, particularly cardiac dysfunction [75]. In mice, overexpression of CUG-binding protein 1 (CUGBP1) leads to various cardiac conditions, such as dilated cardiomyopathy and heart failure, alongside the reactivation of embryonic splicing patterns [20,77,92,93]. RBPs typically expressed during fetal development are also detected in the tissues of adults with heart failure, indicating their reactivation and potential role in the progression of cardiac diseases, particularly in the context of diabetes [63]. Alternative splicing, a regulated process during gene expression, allows a single gene to produce multiple mRNA variants by selectively including or excluding specific exons [94]. RBFOX2, a regulator of genes critical for cardiac function through alternative splicing, is consistently underexpressed in various animal models, leading to reduced heart rate, disorganized myofibrils, and the development of heart failure [26,27]. The reduction in quaking (QKI) isoforms, especially in non-diabetic hearts with heart failure, underscores their crucial role in maintaining cardiac function. Decreased QKI-5 levels are linked to doxorubicin-induced heart failure, while increasing its expression has been shown to alleviate heart failure severity by influencing specific circRNAs [79] (Figure 8). Overall, the intricate network of RNA-binding proteins across various molecular pathways highlights their essential role in preserving cardiac health and contributing to the development of cardiac diseases, offering promising opportunities for therapeutic interventions.

## 4. Therapeutic Potential and Clinical Progress of RBPs in Cardiovascular Disease

Recent studies have emphasized the pivotal role of RBPs in the management of cardiovascular diseases, marking a significant advancement in the field [18]. This discovery offers critical insights for developing innovative therapies to mitigate MIRI [35]. Understanding the molecular mechanisms underlying atherosclerotic cardiovascular disease is crucial for identifying new therapeutic targets. Through their sequence-specific interactions with mRNA, RBPs are involved in various functions throughout disease initiation and progression, providing opportunities to replicate atheroprotective effects or inhibit atherogenic RBP-mRNA interactions. These developments have led to the creation of strategies and tools to modulate RBP expression [95,96]. This shift towards precision-targeted molecular mechanisms represents a more refined approach to combating cardiovascular diseases.

QKI-5 and QKI-6 are essential for maintaining vascular cell integrity, and their re-expression could serve as an effective therapeutic strategy. The synergistic overexpression of these two proteins significantly enhances the beneficial properties of vascular cells, underscoring their therapeutic potential in vascular medicine [54]. Additionally, the unique roles of QKI-5 and QKI-6 in cardiovascular disease management highlight the importance of their expression in promoting vascular health. Meanwhile, RBPs regulate the transcriptome during phenotypic transitions in CVDs. Liao and colleagues identified 1148 RBPs, 393 of which are cardiomyocyte-specific and associated with cardiac conditions [97]. These findings further underscore the multifaceted roles of RBPs in cardiovascular health and their potential as therapeutic targets.

Restoring the normal physiological functions of RBPs is a key focus in cardiovascular research, as this insight is vital for developing effective medical interventions. Elevated levels of human antigen R (HuR) are linked to adverse outcomes in coronary artery disease [98], suggesting the potential of RBPs as early diagnostic and prognostic markers in vascular diseases. However, the application of RBPs as biomarkers faces challenges, including their intracellular nature and the technical difficulties of detecting subtle gene expression changes in clinical samples. In atherosclerosis, RBPs can act as both promoters and inhibitors, complicating their evaluation [99]. To effectively utilize RBPs as diagnostic tools, advanced analytical techniques and a deeper understanding of their roles in cardiovascular health are required. Overcoming these obstacles could greatly improve the precision and effectiveness of atherosclerosis management.

Targeting the functional transcriptome with small molecules presents a novel strategy in therapies involving RBPs [96]. These compounds can interfere with RBP-RNA interactions, suppress RBP functions, and potentially trigger RBP degradation. For example, KH-3 disrupts the interaction between HuR and AU-rich elements (HuR-ARE) at low concentrations through competitive inhibition, thereby impeding HuR function [100]. In a pressure overload-induced hypertrophy mouse model, KH-3 reduced cardiac hypertrophy and improved cardiac function [101]. Additionally, siRNA targeting C-C Motif Chemokine Receptor 2 (CCR2) has been shown to significantly reduce inflammation in atherosclerotic mice [102]. The clinical application of the CCR2 antagonist MLN1202 (humanized monoclonal antibody) is also associated with a significant decrease in high-sensitivity C-reactive protein levels in individuals at high risk for atherosclerotic cardiovascular disease [103]. These strategies underscore the complexity of RBP pathways and their therapeutic potential in indirect regulation. RBPs are generally categorized into two types: pathogenic, which are harmful, and protective, which are beneficial. Therapeutic strategies should focus on replicating the beneficial effects of protective RBPs or inhibiting the harmful effects of pathogenic RBPs to restore health and prevent disease [104]. Furthermore, the sequence-specific binding capabilities of each RBP offer opportunities for therapeutic development, including the creation of drugs that target specific RBPs or RBP-RNA interactions [105,106]. Emerging RNA-based therapies, such as antisense oligonucleotides (ASO), small interfering RNAs (siRNAs), and CRISPR/Cas systems, can inhibit, degrade, or enhance the expression of specific RNA targets, thereby influencing mRNA splicing or translation [105,106]. These diverse therapeutic approaches highlight the complexity and significance of RNA in biological processes and disease mechanisms, providing numerous potential targets for innovative therapies.

## 5. Discussion

The use of antisense oligonucleotides, siRNA, and inhibitors targeting RBPs represents a rapidly advancing approach in the management of atherosclerotic cardiovascular diseases. The pivotal CANTOS study demonstrated that reducing vascular inflammation significantly decreases the incidence of subsequent heart attacks and strokes, thereby addressing a crucial aspect of residual risk [107]. Nonetheless, the ongoing risk of inflammation-related cardiovascular events remains, likely due to the continuous production of cytokines such as interleukin-6 (IL-6) and interleukin-18 (IL-18) [108]. This highlights the necessity for continued research to identify and clarify the key regulatory molecules that influence gene expression relevant to atherosclerosis. Given the central role of vascular inflammation in the progression of atherosclerosis, therapies targeting RBPs are increasingly being developed to modulate this inflammatory response, potentially leading to improved treatment options for atherosclerotic cardiovascular diseases. Addressing these knowledge gaps is crucial to fully harness the therapeutic potential of RBPs. Future research should focus on further exploring the mechanisms by which RBPs influence cardiovascular diseases, including their interactions with other cellular components, the regulation of their activity, and their role in disease pathogenesis. With this understanding, more targeted therapies can be developed to modulate specific RBPs, potentially resulting in more effective treatments for various cardiovascular diseases.

## 6. Conclusions and Outlook

RBPs are pivotal in cardiovascular biology, influencing numerous RNA-related processes such as alternative splicing, polyadenylation, RNA editing, and RNA modification. These processes profoundly impact gene expression, leading to the production of proteins with distinct functions that affect cardiovascular health. RBPs play crucial roles in myocardial injury, arrhythmias, myocardial infarction, and heart failure, making them promising therapeutic targets. Modulating RBPs could potentially improve myocardial function or prevent arrhythmias. Although the development of RBP-targeted therapies is still in its early stages, preliminary studies in animal models suggest significant therapeutic potential. However, the path to clinical application faces challenges, including limited clinical evidence linking RBPs to cardiovascular events and an incomplete understanding of their mechanisms in different cell types and disease contexts. Future research should focus on elucidating these mechanisms and interactions to develop more targeted therapies, ultimately leading to more effective treatments for cardiovascular diseases.

## Figures and Tables

**Table 1:** Roles of RBPs in cardiovascular RNA regulation. Protein amino acid content and molecular weight data are from the UniProt database: “https://www.uniprot.org/”(accessed on 19 August 2024). VSMC: vascular smooth muscle cell.

RBPs	Amino Acids/aa	Mass/Da	Expression or Activity in Cardiovascular Diseases	Function	Targets	Organism	Refs.
MBNL1	343	36,551	Increased	Regulates VSMC proliferation and differentiation	Not explicitly mentioned, but involved in miR-30b-5p interaction	Homo sapiens	[19]
PCBP2	335	35,347	Decreased	Inhibits hypertrophy	GPR56 mRNA	Homo sapiens, Mus musculus	[16]
CELF1	486	52,107	Decreases in adult heart; increases during heart development and in response to cardiac stress	Regulates alternative splicing	Not explicitly mentioned	Mus musculus	[20]
SF1	111	11,695	Increased	Regulation of steroidogenesis	Steroidogenic enzymes	Homo sapiens	[21]
ZRSR2	462	54,905	Increased	Splicing	Not explicitly mentioned	Branchiostoma lanceolatum	[22]
SRSF4	489	55,979	Decreased	Inhibits hypertrophy	GAS5 (growth arrest-specific 5)	Mus musculus	[23]
SRSF5	55	6205	Increased	Facilitates production of p19 H-RAS	p19 H-RAS	Homo sapiens	[24]
CIRP	172	18,648	Increases under chemotherapy-induced stress	Inhibits apoptosis	OGFR mRNA	Homo sapiens	[25]
RBFOX2	449	47,330	Decreases under pressure overload-induced heart failure	Regulates splicing	Exon of fxr1	Mus musculus	[26,27]
FTO	505	58,282	Decreases in failing mammalian hearts and hypoxic cardiomyocytes	Regulates cardiac contractility	SERCA2A, MYH6/7, RYR2	Homo sapiens	[28]
METTL3	580	64,616	Increases in response to hypertrophic stimulation	Catalyzes m^6^A methylation, promoting cardiac hypertrophy	mRNAs encoding kinases involved in the MAPK pathway, such as SERCA2A, MYH6/7, and RYR2.	Mus musculus	[29]

**Table 2:** Roles of RBPs in cardiac disease. Protein amino acid content and molecular weight data are from the UniProt database: “https://www.uniprot.org/”(accessed on 19 August 2024). MIRI: myocardial ischemia-reperfusion injury; VSMC: vascular smooth muscle cell; AF: atrial fibrillation; MI: myocardial infarction; HF: heart failure; DM1: myotonic dystrophy type 1.

RBPs	Amino Acids/aa	Mass/Da	Expression or Activity in Cardiac Diseases	Function	Targets	Study Model	Refs.
MIRI							
ELAVL1	326	36,153	Upregulated	Promotes ferroptosis, modulates autophagy	Beclin-1, FOXC1	Mus musculus	[50]
SRSF10	262	31,301	Downregulated	Regulates splicing of key muscle genes	MEF2A, CASQ2, TTN	Homo sapiens	[51]
QKI-5	341	37,671	Downregulated	Enhances ischemic tolerance, reduces apoptosis	FOXO1 pathway	Mus musculus	[52]
cardiac arrhythmias							
QKI-6	341	37,671	Downregulated	Regulates splicing of HDAC7, promotes VSMC differentiation	HDAC7, SM22	Homo sapiens	[53,54]
CIRP	172	18,607	Upregulated	Prevents AF onset by posttranscriptionally regulating ion channels	Kv1.5 and Kv4.2/4.3 ion channels	Mus musculus	[55]
MI							
RBM15	962	105,722	Upregulated	Stabilizes m6A modification, improves cardiac function	NEDD8	Mus musculus	[56]
IGF2BP2	592	65,584	Upregulated	Modulates RNA localization, enhances stress response	Sarcomeric and mitochondrial proteins	Mus musculus	[57]
LIN28A	209	22,720	Upregulated	Inhibits cardiac remodeling and apoptosis, improves cardiac function	UPF1	Mus musculus	[58,59]
RbFox1	396	42,678	Downregulated	Regulates the alternative splicing of Cav1.2 channels	Exon 9* and exon 33 of the Cav1.2 calcium channel gene	Mus musculus	[60]
RbFox2	449	47,330	Downregulated	Regulates the alternative splicing of Cav1.2 channels	Exon 9* and exon 33 of the Cav1.2 calcium channel gene	Mus musculus	[60]
SRSF3	164	19,330	Downregulated	Prevents mRNA decapping involved in cardiac contraction	mTOR mRNA	Mus musculus	[61]
HF							
Cpeb4	729	80,122	Dynamically expressed in cardiomyocytes	Regulates cardiac remodeling	Zeb1, Zbtb20	Mus musculus	[62,63]
LIN28A	209	22,720	Upregulated	Regulates cardiac remodeling genes, alleviates cardiac hypertrophy, promotes cardiac regeneration	lncRNA H19, let-7 miRNA	Mus musculus	[64,65,66,67,68]
FUS	518	52,673	Downregulated	Regulates cardiomyocyte apoptosis	circFndc3b, KCNQ1OT1	Mus musculus	[69,70]
RBM20	1199	130,124	Upregulated	Splicing regulation	cTTN1, titin	Mus musculus	[71,72]
YTHDF2	579	62,280	Upregulated	Suppresses cardiac hypertrophy via m^6^A-dependent regulation of Myh7 mRNA	Myh7 mRNA	Mus musculus	[73]
RBM25	843	100,185	Upregulated	Mediates abnormal mRNA splicing of SCN5A, reducing Na+ channel current	SCN5A mRNA	Homo sapiens	[74]
LUC7L3	118	13,445	Upregulated	Mediates abnormal mRNA splicing of SCN5A, reducing Na+ channel current	SCN5A mRNA	Homo sapiens	[74]
TDP-43	414	44,740	Upregulated	Promotes cardiac muscle degeneration	Not explicitly mentioned	Homo sapiens	[75,76]
CUGBP1	486	52,107	Upregulated	Disrupts splicing in DM1	Not explicitly mentioned	Mus musculus	[77]
RBFOX2	449	47,330	Downregulated	Regulates alternative splicing	Rho GTPase cycling genes	Mus musculus	[78]
QKI-5	341	37,671	Downregulated	Inhibits doxorubicin-induced cardiac damage	Ttn (titin), Fhod3, Strn3	Mus musculus	[79]

## Data Availability

No new data were created in this work.
